# Pharmacometrics modeling and simulation to assist phenobarbital dose optimization in dogs

**DOI:** 10.3389/fvets.2025.1644003

**Published:** 2025-09-03

**Authors:** Silvana Alvariza, Manuel Ibarra, Natalia Guevara, Cecilia Maldonado, Marta Vázquez, Gimena Feijoó, Gonzalo Suárez

**Affiliations:** ^1^Department of Clinical and Veterinary Hospital, Faculty of Veterinary, Pharmacology and Therapeutics Unit, Universidad de la República, Montevideo, Uruguay; ^2^Pharmaceutical Sciences Department, Therapeutic Drug Monitoring Service, Faculty of Chemistry, University Hospital, Montevideo, Uruguay

**Keywords:** phenobarbital, population pharmacokinetics, therapeutic drug monitoring, model-informed precision dosing, canine epilepsy

## Abstract

**Introduction:**

Phenobarbital (PB) remains the first-line treatment for canine epilepsy due to its efficacy, affordability, and favorable pharmacokinetics. However, its narrow therapeutic index and substantial interindividual variability necessitate therapeutic drug monitoring (TDM) and individualized dosing. This study aimed to develop and validate a population pharmacokinetic (popPK) model of PB in dogs to support model-informed precision dosing (MIPD) in clinical practice.

**Methods:**

A total of 121 serum samples from 100 dogs receiving PB monotherapy at steady state were used to build the model. An external dataset comprising 53 samples from 50 dogs was used for validation. Modeling was performed using nonlinear mixed-effects (NLME) techniques in MonolixSuite 2023R1. Covariate analysis included age, sex, and body weight (WT). Model performance was assessed using goodness-of-fit plots, prediction-corrected visual predictive checks (pcVPC), and calculation of mean error (ME), mean relative error (MRE), and root mean square error (RMSE). Monte Carlo simulations were conducted to evaluate the probability of target attainment (PTA) under different dosing regimens.

**Results:**

A one-compartment model with autoinductive clearance (CL) best described PB pharmacokinetics. WT and age were significant covariates on apparent clearance (CL/F). The model accurately predicted PB concentrations in the external dataset (ME = −0.08 mg/L, MRE = 0.04%, RMSE = 2.04%). Simulations identified optimal dosing regimens stratified by age and WT, including recommendations for loading doses to accelerate attainment of therapeutic concentrations.

**Discussion:**

The validated popPK model enables individualized PB dosing in dogs, accounting for variability in WT and age. This approach supports the implementation of MIPD in veterinary practice and may improve therapeutic outcomes while minimizing toxicity.

## 1 Introduction

Pharmacometrics, also referred to as “the science of quantitative pharmacology” (1), is a multidisciplinary field that develops and applies mathematical and statistical models to characterize variability in drug exposure, response, and disease progression. It supports data-driven decision-making in both drug development and clinical pharmacotherapy. This discipline integrates principles of pharmacokinetics (PK), pharmacodynamics (PD), pharmaceutical sciences, therapeutics, and pathophysiology, using mathematical and computational tools to describe and predict drug–organism interactions and clinical outcomes (2). Such models provide a rational framework to anticipate variability in drug exposure and response across different dose levels and populations. The use of computational tools enhances the value of data obtained from clinical trials, therapeutic drug monitoring, and pharmacovigilance (20).

Phenobarbital (PB) is the first-line treatment for seizures in canine epilepsy due to its efficacy, low cost, and favorable pharmacokinetic properties, which allow dosing every 12–24 h, unlike other classic anticonvulsants such as phenytoin or carbamazepine (3). Following oral administration in dogs, PB is rapidly and almost completely absorbed (bioavailability >85%), reaching peak plasma concentrations between 4 and 8 h post-dose. Approximately 50% of PB binds to plasma proteins. Its primary elimination pathway is hepatic enzymatic biotransformation, with about 33% excreted unchanged in the urine (4). PB is also an auto-inducer of the microsomal CYP450 enzyme complex, which leads to a progressive reduction in its elimination half-life with chronic use (5). According to Thurman et al. (21), the mean elimination half-life is 46.3 ± 11.3 h after a single dose and 29.3 ± 4.6 h at steady state, typically reached after 3 weeks of daily dosing.

Due to its narrow therapeutic index (15–45 mg/L) and significant pharmacokinetic interindividual variability observed in dogs, therapeutic drug monitoring (TDM) of PB is strongly recommended to optimize dosing, maximizing the probability of therapeutic efficacy while minimizing the risks of toxicity including sedation, lethargy, ataxia and hepatotoxicity (6, 7).

Traditionally, therapeutic drug monitoring relies on trough concentration, which provides limited information for individual pharmacotherapeutic interpretation and clinical outcome prediction. Nonlinear mixed effects (NLME) models implemented in pharmacometrics analyses allow the use of sparse data in the development of tools based on the population approach. In this context, population pharmacokinetic parameters can be estimated from sparse observations performed in patients belonging to the same population, and sources of interindividual and interoccasion variability can be recognized and described in a quantitative fashion. Furthermore, population pharmacokinetic models can be employed in the clinical setting to obtain maximum *a posteriori* estimates of individual parameters using sparse observations from the patient and the *a priori* distribution provided by the model. Bayesian forecasting is increasingly implemented within the context of model-informed precision dosing (MIPD) (8, 9).

Previous population pharmacokinetic studies in humans have identified several factors that significantly influence phenobarbital C, including total body weight, body surface area, and concomitant use of other antiepileptic drugs (10). To date, however, such models have not been applied to support dose optimization of phenobarbital in dogs. In this study, we aimed to develop a population pharmacokinetic model for phenobarbital in canine patients, evaluate current dosage regimens, and propose individualized dosing strategies based on patient-specific characteristics. Our goal was to provide a practical tool to support evidence-based dosing adjustments in clinical veterinary practice.

## 2 Materials and methods

### 2.1 Patients and data collection

A total of 121 serum samples were obtained from 100 client-owned dogs receiving phenobarbital monotherapy at steady state, defined as at least 20 days after treatment initiation. These data were used to develop the popPK model.

An independent validation dataset, consisting of 53 serum samples from 50 dogs, was also available. Dogs included in both datasets varied in age, body weight, sex, and neuter status; no breed restrictions were applied, and breed was not considered as a covariate in the analysis. All samples were collected as part of routine therapeutic drug monitoring with informed owner consent, under a protocol approved by the institutional Animal Use Ethics Committee.

Dogs received commercial oral phenobarbital tablets (100, 40 or 15 mg), administered according to the prescribing veterinarian’s instructions. Dosing was typically based on whole or halved tablets, reflecting standard clinical practice.

To assess demographic comparability between the training and validation populations, sex distribution was evaluated using the chi-square test, and continuous variables (age and BW) were compared using unpaired t-tests. No statistically significant differences were observed (*p* > 0.05), indicating that the two populations were demographically comparable. A summary of baseline characteristics is presented in Table 1.

**Table 1:** Demographic characteristics of the canine population undergoing monotherapy with phenobarbital at steady state.

Population characteristics	Training group	Validation group
Number of patients (*n*)	100	50
Number of observations (*n*)	121	53
Sex (male/female)^1^	57/43	30/20
Age (mean ± SD, years)^2^	5.4 ± 3.5	4.8 ± 2.8
BW (mean ± SD, kg)^2^	21.3 ± 14.0	21.6 ± 12.0

Statistical test performed: Chi-square test of independence^1^, *t*-test^2^, *p* > 0.05. BW, weight.

### 2.2 Phenobarbital determination

Phenobarbital serum concentrations were determined using the ARCHITECT® iPhenobarbital assay (Abbott), a chemiluminescent microparticle immunoassay (CMIA) performed on the ARCHITECT i1000SR analyzer. The assay’s validated analytical range is 1.1–80.0 μg/mL, with a lower limit of quantification (LLOQ) of 1.1 μg/mL. Intra- and inter-assay coefficients of variation (CV) were below 4%, and accuracy was within ±10% across the measurement range. Analyses were conducted at the Therapeutic Drug Monitoring Service, Department of Pharmaceutical Sciences, University Hospital, Universidad de la República (Uruguay), in accordance with the manufacturer’s validation standards.

### 2.3 Pharmacokinetic modeling

#### 2.3.1 Structural model

Phenobarbital serum concentrations were analyzed using NLME implemented in MonolixSuite 2023R1 (Lixoft, a Simulations Plus Inc., company) to estimate the typical CL/F of PB and to perform the covariate analysis. Due to the sparse nature of the dataset, a one-compartment disposition model was selected. The absorption rate constant (ka) and the apparent volume of distribution (Vd/F) were fixed at 0.61 h^−1^ and 20.0 L, respectively, based on previously published data (3, 5, 11). Both linear and autoinductive CL models were evaluated.

Interindividual variability (IIV) in CL/F was described using a log-normal distribution, as shown in Equation 1:


(1) $$\mathrm{CL}/F_{i}=\mathrm{CL}/F_{\mathrm{pop}}*e^{\eta_{i}}$$

Where $\mathrm{CL}/F_{i}$ and $\mathrm{CL}/F_{\mathrm{pop}}$ represent the ith individual estimate and the typical (population) parameter, respectively. The random effects $\eta_{i}$ describe the ith individual’s deviation from $\mathrm{CL}/F_{\mathrm{pop}}$ with a normal distribution: $\eta_{i}\sim N(0,\omega^{2}),$ being $\omega^{2}$ the IIV variance.

Residual unexplained variability was modeled using an additive error model, as described in Equation 2:


(2) $$C_{i,j}=C_{\mathrm{pre}d_{i,j}}+a*\varepsilon_{i,j}$$

In this equation, *C_ij_* represents the observed concentration for the *i*th subject at time *j*, *Cpred_ij_* is the corresponding model-predicted concentration, *a* is the estimated additive residual error, and *ε_ij_* is a standard normal random variable with mean 0 and variance 1.

#### 2.3.2 Model evaluation

The model-building process was guided by diagnostic metrics and graphical diagnostics. The corrected Bayesian Information Criterion (BICc) computed from the estimated log-likelihood was used to optimize model parsimony, assessing the tradeoff between data fit and model complexity. Uncertainty in parameter estimation, calculated with the Fisher Information Matrix in Monolix® via linearization and expressed as relative standard error (RSE) was also considered as an essential diagnostic, along with plausibility of estimates. Basic goodness-of-fit plots included observed concentrations versus individual and population predictions, residuals plotted against time and predicted concentrations, and the distributions of both residuals and random effects. In addition, simulation-based diagnostics were performed, including prediction-corrected visual predictive checks (pcVPC) and normalized prediction distribution errors (NPDE).

##### 2.3.2.1 Covariates analysis

The impact of age (years), sex, and body weight (WT), in kg on CL/F (L/h) was evaluated once the base structural model was developed.

Continuous covariates were incorporated using a power model with median centering, as described in Equation 3:


(3) $$\mathrm{CL}/F_{i}=\mathrm{CL}/F_{\mathrm{pop}}*{\left(\frac{{\mathrm{COV}}_{i}}{\mathrm{CO}V_{\mathrm{pop}}}\right)}^{\beta_{\mathrm{cov}}}e^{\eta_{\theta_{i}}}$$

Where ${\mathrm{COV}}_{i}$ is the individual value of the continuous covariate for the ith animal, $\mathrm{CO}V_{\mathrm{pop}}$ the population median value (use for centering), $\beta_{\mathrm{cov}}$ represents the estimated effect of the covariate on CL/F ηᵢ the random effect representing unexplained IIV.

Categorical covariates such as sex a categorical covariate, were evaluated according an exponencial model, as shown in Equation 4:


(4) $$\mathrm{CL}/F_{i}=\mathrm{CL}/F_{\mathrm{pop}}*e^{\beta_{\mathrm{cat}}*{\mathrm{sex}}_{i}}\;e^{\eta_{\theta_{i}}}$$

Where $\beta_{\mathrm{sex}}$ SEX_i_ is coded as 1 for males and 0 for females, $\beta_{\mathrm{sex}}$ s the estimated effect associated with sex, and η_i_ is the random effect accounting for IIV not explained by the covariate.

A forward selection method guided by graphical and statistical diagnostics was used to build the final model. The likelihood ratio test (LRT) and the Wald test were implemented to assess the statistical significance of each evaluated covariate relation. Covariate effects were retained in the model during the forward selection if its inclusion resulted in a reduction of the objective function value (OFV: −2*log-likelihood) by at least 3.84 units (*p* < 0.05) for the likelihood ratio test or LRT with one degree of freedom (12), and if the estimated parameter was significantly different than 0 (*p* < 0.05 in the Wald test). A backward deletion was then implemented to refine the full model based on the LRT, retaining significant effects with *p* < 0.01 (6.63-unit change in the objective function).

A stepwise approach was used to build the final model, beginning with forward selection and followed by backward elimination. The inclusion of a covariate was retained during forward selection if it reduced the objective function value (OFV = −2 × log-likelihood) by at least 3.84 units (*p* < 0.05) for the Likelihood Ratio Test (LRT) with one degree of freedom, and if the estimated parameter was significantly different from zero (*p* < 0.05 in the Wald test). In the backward elimination step, covariates were retained if their removal increased the OFV by at least 6.63 units (*p* < 0.01 for the LRT). Graphical and statistical diagnostics supported the selection process throughout.

#### 2.3.3 External model validation

The validation dataset was used to assess the predictive performance of the final model. Both the *a priori* prediction (i.e., model predictions obtained for each individual using covariate data only) and the prediction obtained after Bayesian updating of pharmacokinetic parameters (i.e., after estimating individual parameters using individual characteristics and observations) were evaluated with goodness of fit plots and metrics. Mean error (ME, Equation 4), mean relative error (MRE, Equation 2), and relative root mean squared error (RMSE, Equation 3) were calculated to assess prediction bias and precision, as shown in Equations 5–7:


(5) $$\mathrm{ME}\;(\frac{\mathrm{mg}}{L})=\frac{\sum (\text{Cpred}-\text{Cobs})}{n}$$

(6) $$\mathrm{MRE}\;(\%)=\frac{\sum (\frac{\text{Cpred}-\text{Cobs}}{\text{Cobs}})}{n}.100$$

(7) $$\text{RMSE}(\%)=\sqrt{\frac{\sum {\left(\frac{\text{Cpred}-\text{Cobs}}{\text{Cobs}}\right)}^{2}}{n}.100}$$

Where *Cpred* is the concentration predicted by the model, *Cobs* is the observed concentration and *n* is the number of observations.

### 2.4 Simulation assessment for different dose regimens

Monte Carlo simulations were performed using the final model in Simulx 2023R1 (Lixoft SAS, a Simulations Plus company) to evaluate PB pharmacokinetics under various dosing scenarios. A total of 1,000 individual profiles were simulated to explore different dosage regimens and estimate the probability of target attainment (PTA), defined as the proportion of individuals achieving steady-state concentrations within the therapeutic range. Therapeutic concentrations were defined as those between 15 and 45 μg/mL, based on published clinical guidelines (4). A PTA of 90% was considered an acceptable threshold for regimen selection. Additionally, the inclusion of a loading dose was evaluated to accelerate the achievement of therapeutic concentrations.

## 3 Results

A one-compartment model with an elimination process that is enhanced over time due to autoinduction was found to best describe PB concentration – time data. Autoinduction models have been proposed by Smythe et al. (13), and in Hassan et al. (14) to describe drugs that stimulate its own metabolism. In this model, CL/F increases over time as PB induces the expression of metabolic enzymes responsible for its own elimination.

The system is described by the following differential equations:


(8) $$\frac{\mathrm{dAc}}{\mathrm{dt}}=\mathrm{ka}-\frac{CL}{V}\times \mathrm{Enz}.\mathrm{Ac}$$

(9) $$\text{dEnz}=\mathrm{ken}z_{\text{in}}-\mathrm{ken}z_{\text{out}}\times (1-\frac{\mathrm{Cc}}{\mathrm{Cc}+IC_{50}})$$

The time-course of the system is described by two differential equations. Equation 8 models the amount of drug in the central compartment (Ac), which decreases over time due to CL modulated by the level of metabolizing enzymes (Enz). Equation 9 describes the dynamics of the enzyme pool. Although Kenz_in_ and Kenz _out_ are distinguished to reflect the mechanistic interpretation of enzyme synthesis and degradation, the model assumes that both parameters are equal (Kenz_in_ = Kenz_out_ = Kenz_pop), implying steady-state enzyme levels in the absence of drug.

**Figure 1:**
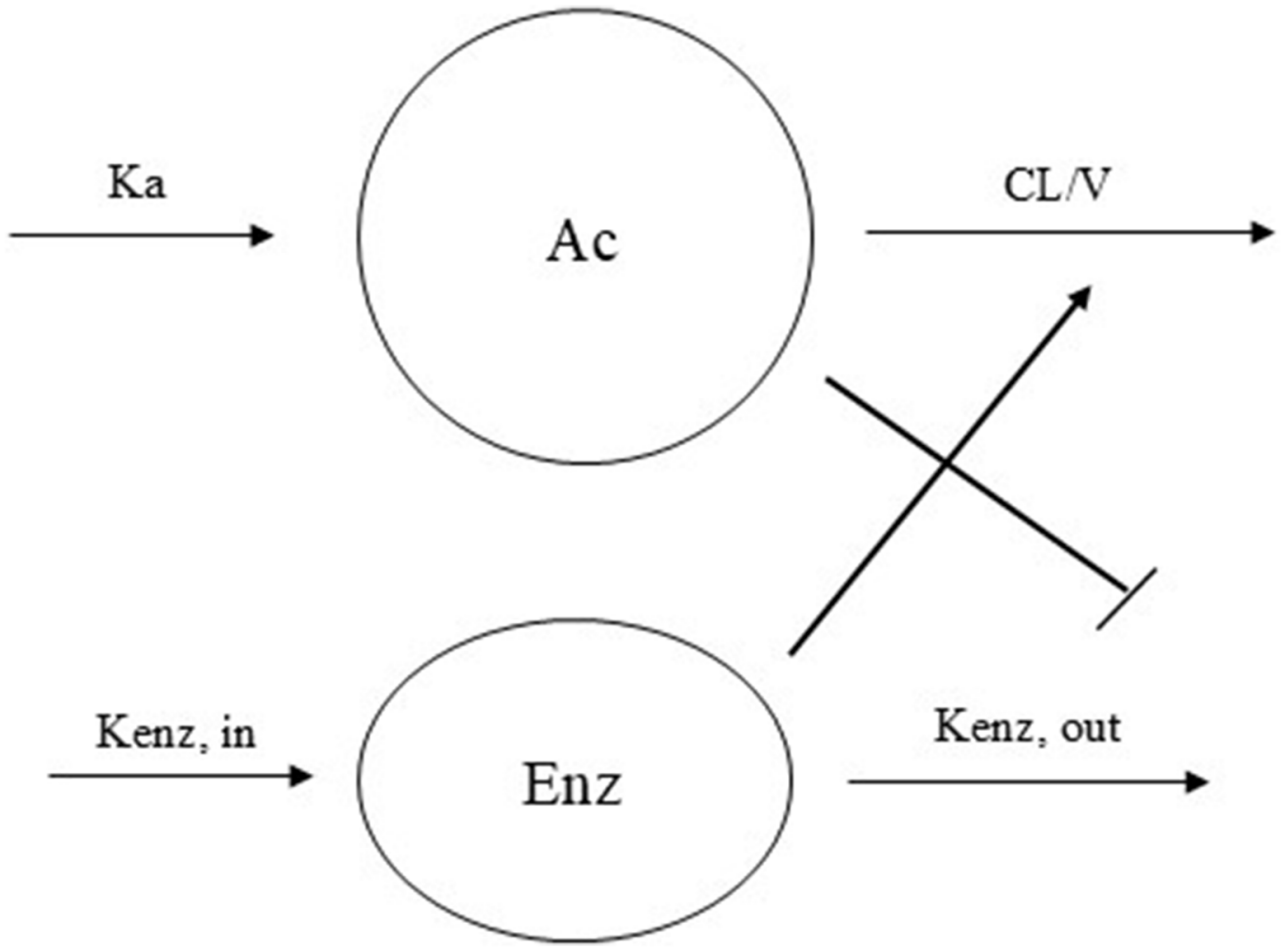
Schematic representation of the autoinduction model with enzyme turn over phenobarbital CL model. Ka = absorption rate constant, CL/V = elimination rate constant, Kenz,_in_ = enzyme production constant rate, Kenz,_out_ = enzyme degradation constant rate.

This simplification allows the autoinductive effect of phenobarbital (PB) to be represented as an inhibition of enzyme degradation, governed by a sigmoidal function in which *IC_50_* represents the PB concentration that reduces enzyme degradation by 50%. As PB concentrations increase, enzyme degradation slows down, resulting in progressive enzyme accumulation over time. Since these enzymes mediate PB metabolism, their accumulation leads to a time-dependent increase in apparent CL/F. This dynamic behavior, captured by the model, aligns with clinical observations of reduced PB exposure over time despite constant dosing (Figure 1).

The apparent volume of distribution (Vd/F) was fixed to a literature value and scaled linearly with WT according to Equation 10.


(10) $$V=\text{Vpop}.(\frac{\mathrm{WT}}{20})$$

Where 20 kg represents the weighted mean WT in the study population.

**Figure 2:**
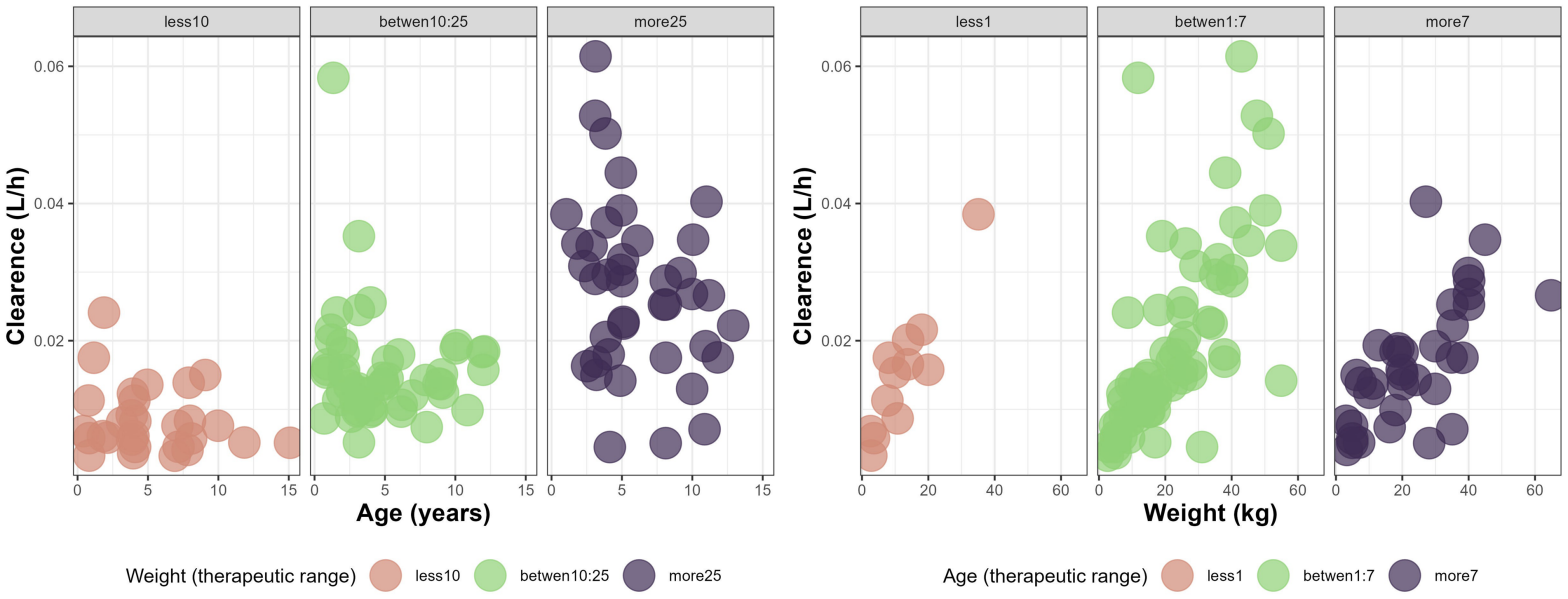
Variations of CL (L/h) with age (years) and weight (kg) in different therapeutic range based on age and weight for the canine population undergoing monotherapy with phenobarbital at steady state.

Both age and WT significantly influenced CL/F, while sex was not a significant covariate (Figure 2, generated using R version 4.5.0 (22)). Incorporating WT into the model reduced the objective function value (OFV) by 62 units. Adding age led to a further decrease of 8 units. CL was significantly influenced by both body weight and age, while sex showed no significant effect. Inclusion of body weight using allometric scaling with a fixed exponent of 0.75 reduced the objective function value (OFV) by 62 units. Adding age as a covariate resulted in an additional reduction of 8 units.

Equation 11 represents the final model equation for CL/F:


(11) $$\mathrm{CL}/F=\text{CLpop}{\left(\frac{\mathrm{WT}}{20}\right)}^{3/4}.{\left(\frac{\mathrm{AGE}}{5}\right)}^{\beta_{\mathrm{AGE}}}$$

Where 20 kg and 5 years correspond to the population median values used for centering.

**Figure 3:**
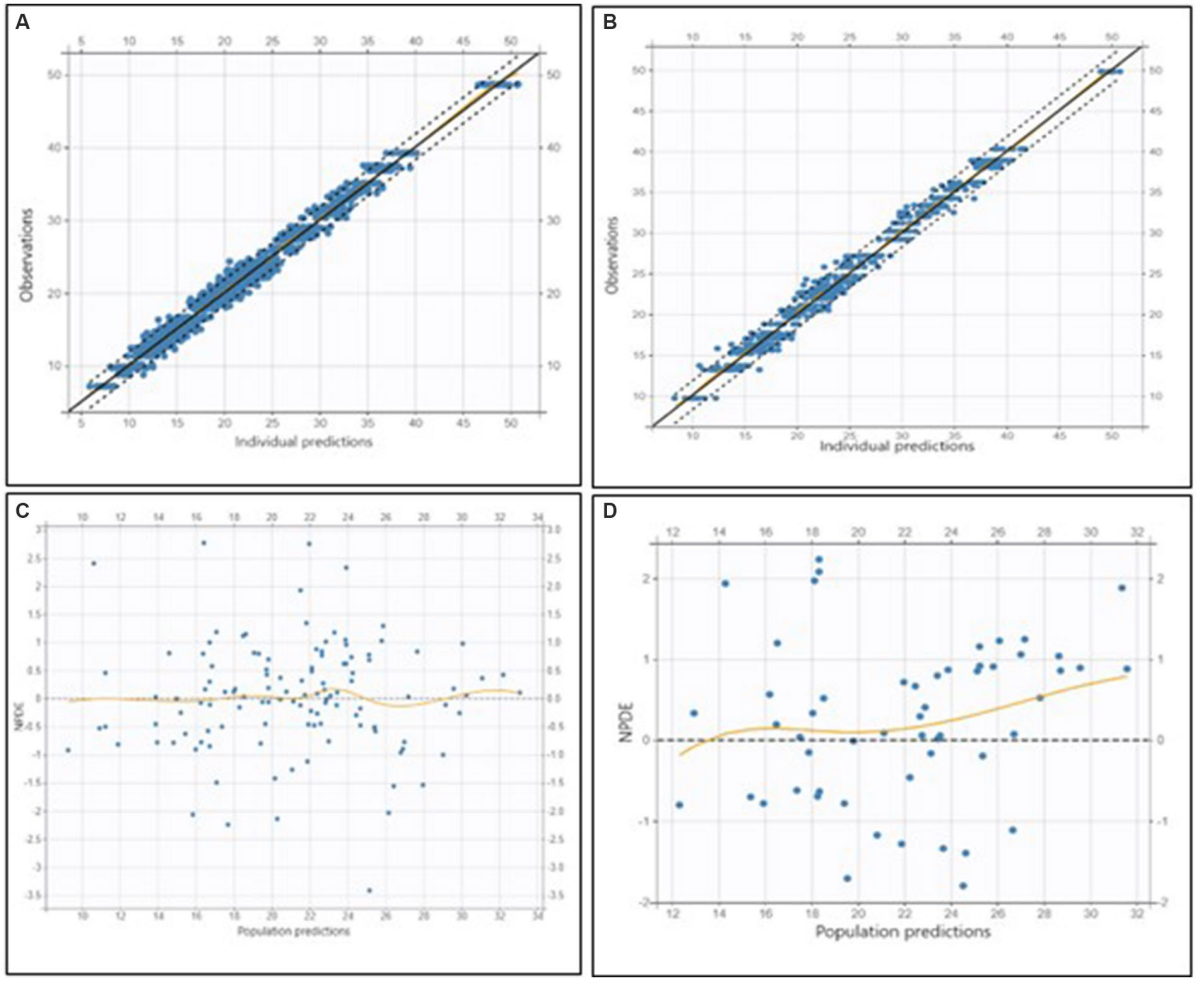
Goodness-of-fit and residual diagnostic plots for the final model applied to the training dataset (left column) and to the external validation dataset (right column) in dogs receiving phenobarbital monotherapy at steady state. **(A)** Top left: Observed concentrations versus individual predictions (OBS vs. PRED) for the training dataset. **(B)** Top right: OBS vs. PRED for the validation dataset. **(C)** Bottom left: Normalized prediction distribution errors (NPDE) versus population predictions for the training dataset. **(D)** Bottom right: NPDE versus population predictions for the validation dataset. In the OBS vs. PRED plots (top row), the black dashed line represents the line of identity, while the dotted lines indicate the 90% prediction interval. The bottom row shows residual diagnostics, with orange loess lines indicating trends in residuals, and the horizontal gray line representing the zero residual level.

The final population pharmacokinetic model characterized the disposition of PB in dogs receiving monotherapy at steady state. The estimated model parameters are summarized in Table 2 and illustrated in Figure 3.

**Table 2:** Estimated population parameters for the canine population undergoing monotherapy with phenobarbital at steady state (final model).

Population parameters	Value	Stochastic approximation	
		S.E.	R.S.E. (%)
ka_pop (h^−1^)	0.6	Fixed	
V_pop (L)	20	Fixed	
CL_pop (L/h)	0.015	0.001	6.26
β_CL_tAGE	−0.15	0.057	37.5
Kenz_pop h^−1^	0.15	0.052	34.5
IC_50__pop (mg/L)	1.77	0.093	5.24

Standard deviation of the random effects				
	Value	CV (%)		
omega_CL	0.28	29.08	0.092	32.1
gamma_CL	0.34	34.72	0.079	23.5
Error model parameters				
a	1.32		0.37	27.7

Population pharmacokinetic parameters of the final model, with standard errors (S.E.) and relative standard errors (R.S.E.%) estimated using the stochastic approximation method. ka_pop, population absorption rate constant; V_pop, population apparent volume of distribution; CL_pop, population apparent clearance; β_CL_tAGE, covariate effect of age on CL/F; Kenz_pop, population rate constant for enzyme turnover; IC_50__pop, population PB half-maximal inhibition of enzyme degradation.

Parameter precision was assessed using the Fisher Information Matrix, and the results indicated reliable estimates.

External validation demonstrated that the model adequately predicted observed concentrations, with a mean error (ME) of −2.10 mg/L, a mean relative error (MRE) of −1.74, and a relative root mean squared error (RMSE) of 24.80% (Figure 3).

**Figure 4:**
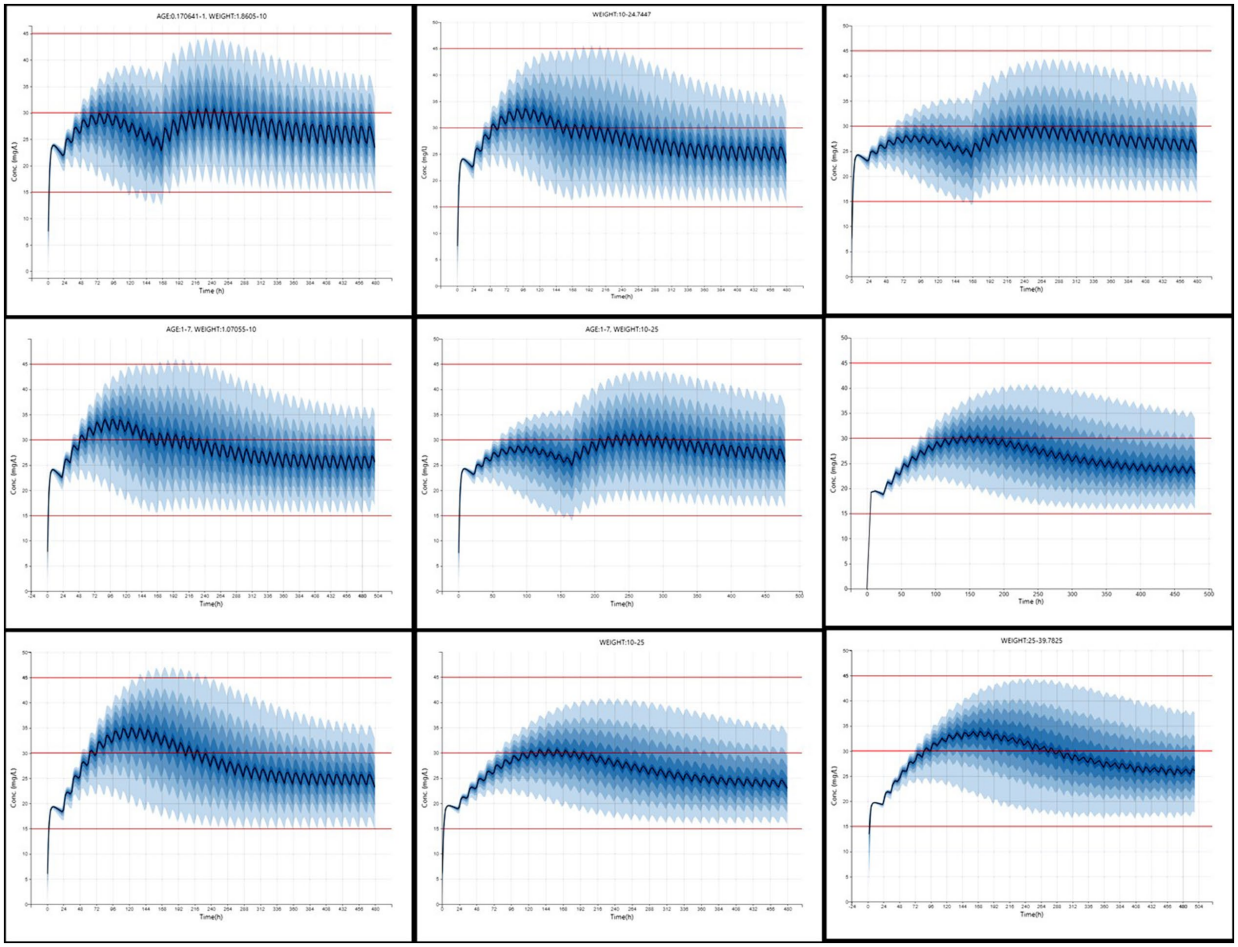
Simplified visual predictive check (VPC) for phenobarbital predose concentrations.

The predictive performance of the final model was explored using a simplified visual predictive check (VPC) adapted in R version 4.5.0 (22), focusing on predose concentrations. Figure 4 presents observed serum concentrations at 12 h post-dose.

**Figure 5:**
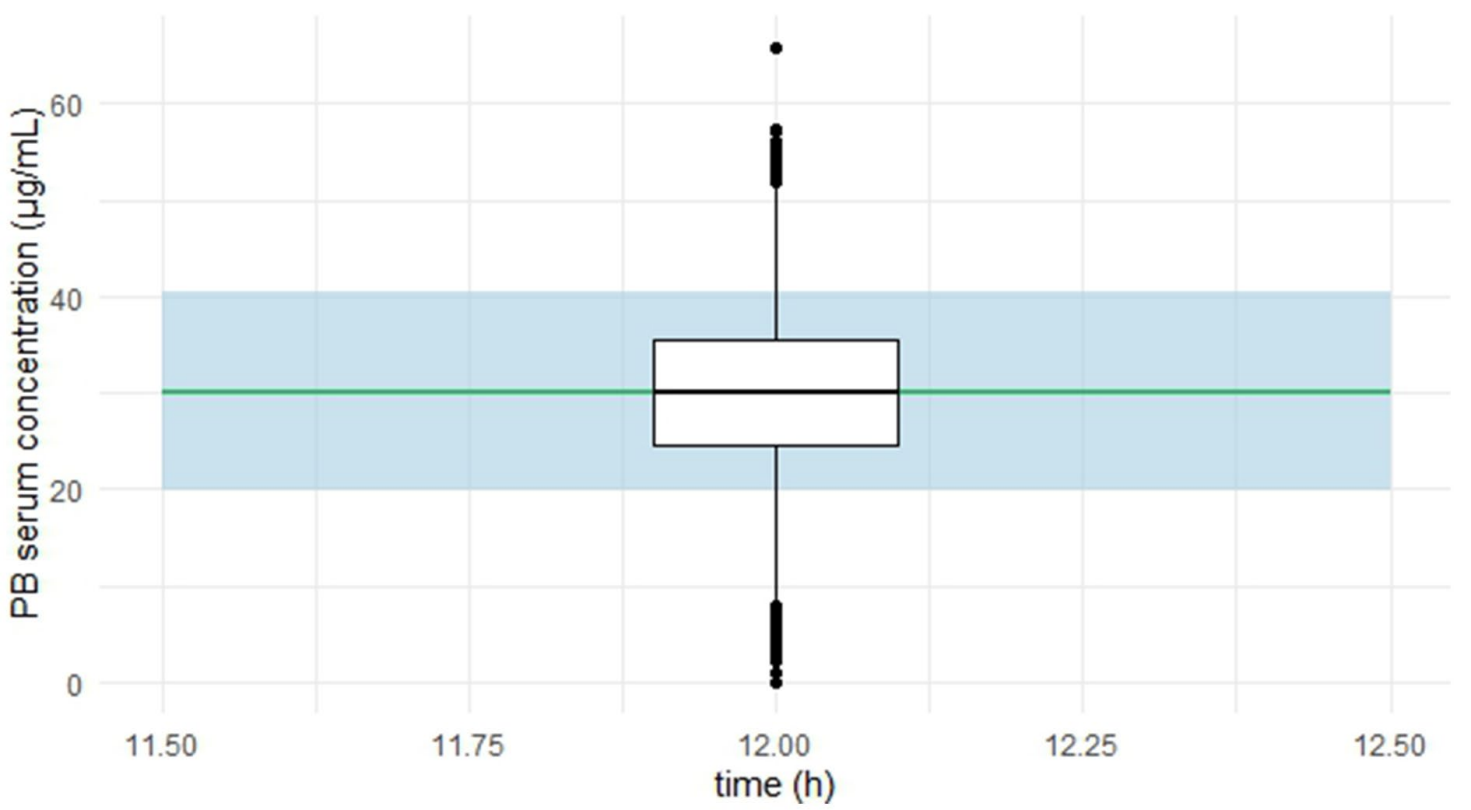
Simulated phenobarbital concentration-time profiles (n = 1,000) across nine age and weight strata in dogs receiving optimized dosing regimens. Each panel represents a subgroup based on weight (kg) and age (years), as defined in Table 3. The solid blue line denotes the median predicted serum concentration, and the shaded area represents the 90% prediction interval. Red horizontal lines indicate thresholds at 15, 30, and 45 μg/mL.

Dosage regimens derived from simulation-based analysis using the final model are summarized in Table 3 and illustrated in Figure 5, highlighting the influence of age and weight on phenobarbital exposure and supporting the rationale for stratified dosing. The resulting recommendations aim to guide individualized phenobarbital therapy in dogs to rapidly and consistently achieve therapeutic concentrations.

**Table 3:** Dosage regimen recommendations to attain a therapeutic range of phenobarbital based on the age and weight of the individual dogs.

Weight (kg)		Age (years)		
		≤1	1–7	≥7
Less than 10	Loading dose.	25 mg/kg single dose.	25 mg/kg single dose.	20 mg/kg/24 h from 0 to 48 h.
	Progression dose.	5 mg/kg/12 h from 24 h for 5 days	5 mg/kg/12 h from 24 h for 5 days	3 mg/kg/12 h for 5 days
	Maintenance dose.	8 mg/kg/12 h	6 mg/kg/12 h	5 mg/kg/12 h
10 to 25	Loading dose.	25 mg/kg single dose	25 mg/kg single dose.	20 mg/kg single dose
	Progression dose.	4 mg/kg/12 h from 24 h for 5 days	3 mg/kg/12 h from 24 h for 5 days	–
	Maintenance dose.	6 mg/kg/12 h	5 mg/kg/12 h	4 mg/kg/12 h
More than 25	Loading dose.	25 mg/kg single dose.	20 mg/kg single dose	20 mg/kg single dose
	Progression dose.	3 mg/kg/12 h from 24 h for 5 days	–	–
	Maintenance dose.	5 mg/kg/12 h	3 mg/kg/12 h	3 mg/kg/12 h

## 4 Discussion

Variability in drug disposition, both between and within individuals, poses a major challenge in the treatment of chronic conditions such as epilepsy. In this study, a popPK model was developed to characterize PB concentrations in dogs receiving monotherapy, based on real TDM data. The model demonstrated adequate predictive performance, as confirmed by external validation, and identified relevant covariates that explain a significant portion of the observed variability, supporting its clinical applicability.

The selected structural model was a one-compartment model with an elimination process subject to autoinduction, in which CL/F increases progressively over time. This type of model, previously proposed by Smythe et al. (13) and Hassan et al. (14), simulates the upregulation of metabolizing enzymes as a result of their degradation being inhibited by the drug. The IC_50_ parameter represents the PB concentration that reduces enzyme degradation rate by 50% and plays a central role in capturing the system’s dynamics and adequately simulating progressive enzyme induction. This mechanism helps explain the plasma concentration profiles observed after treatment initiation or dose adjustments with PB. Initially, concentrations rises due to drug accumulation, but then progressively decline as PB induces the expression of metabolizing enzymes. This increase in CL stabilizes over time, such that at steady state, plasma concentrations remain at levels lower than those reached during the first days of treatment. This behavior contrasts with linear pharmacokinetics, where concentrations gradually rise until stabilizing at a level proportional to the administered dose, typically within 5–7 elimination half-lives. The autoinductive structural model captured this temporal pattern accurately, whereas linear models, though statistically acceptable, failed to represent the observed dynamic or provide a mechanistic explanation. Moreover, the autoinductive model yielded better fit metrics, supporting its selection as a more realistic predictive tool. Importantly, the use of a one-compartment model is consistent with both biological plausibility and previous evidence. A systematic review by Methaneethorn and Leelakanok (15), which evaluated 18 pharmacokinetic studies of phenobarbital in humans, found that all employed one-compartment models, even in the presence of rich sampling designs. This reinforces the empirical and translational robustness of our structural model selection.

In addition, chronic administration of antiepileptic drugs—including PB, phenytoin, and carbamazepine—has been associated with increased expression of efflux transporters such as P-glycoprotein (Pgp) in the brains of rats after 60 days of treatment (16). Efflux transporters are membrane proteins widely distributed in tissues, acting in concert with metabolic enzymes to regulate intracellular drug concentrations by actively exporting their substrates. Pgp is constitutively expressed in the liver, bile canaliculi, intestines, kidneys, blood–brain barrier, and other tissues (17) and significantly influences PB pharmacokinetics by limiting absorption and enhancing biliary and renal elimination. Alvariza et al. (18) further demonstrated that Pgp overexpression occurs in a concentration-dependent manner across multiple tissues, contributing to increased CL/F through a combined effect of enzyme induction and enhanced excretory CL.

The final model also incorporated significant effects of WT and age on CL/F. WT was accounted for using allometric scaling with a fixed exponent of 3/4, in line with well-established physiological principles. Age showed a negative effect on CL, consistent with age-related decline in renal function. In this regard, Laroute et al. (19) reported a 34% decrease in glomerular filtration rate between young and elderly Beagle dogs—a relevant finding given that approximately 25% of PB elimination occurs via renal excretion.

To assess the model’s clinical utility, Monte Carlo simulations were performed in Simulx® under different dosing schemes. A PTA ≥ 90% was considered an acceptable threshold for regimen selection, defined as the proportion of individuals achieving steady-state concentrations within the recommended therapeutic range (15–45 μg/mL). The simulations also included evaluation of loading doses to accelerate attainment of effective concentrations. According to the model, PB concentrations tend to stabilize after approximately 15 days of continuous treatment.

The results showed that dogs weighing less than 10 kg—and particularly those under 1 year of age—require higher doses to reach therapeutic levels, whereas older dogs may require lower doses. In clinical practice, PB is typically prescribed at doses ranging from 2.5 to 5.5 mg/kg, with body weight being the primary factor guiding dose selection Podell et al. (4). However, our findings suggest that age also plays a relevant role in drug disposition and should be considered when individualizing therapy, especially in very young or elderly dogs.

Finally, although other factors such as breed or reproductive status may be clinically relevant, these were not included as covariates due to limitations in the available data and the objective of developing a broadly applicable model for diverse canine populations.

In summary, this study demonstrates the value of pharmacometric tools in optimizing veterinary pharmacotherapy. The proposed model enables individualized dosing strategies based on patient-specific characteristics such as weight and age, which may improve the safety and effectiveness of antiepileptic therapy with phenobarbital.

## 5 Conclusion

This study presents a population pharmacokinetic model that successfully characterizes the disposition of phenobarbital (PB) in dogs receiving monotherapy, incorporating key covariates such as body weight and age. The model not only demonstrated good predictive performance but also mechanistically captured the time-dependent changes in PB CL due to autoinduction. Based on this model, individualized dosing regimens were proposed, allowing for more precise and clinically relevant dose adjustments from the outset and throughout therapy. While current clinical practice primarily considers body weight when selecting PB doses, our findings highlight the importance of also accounting for age, especially in puppies and senior dogs—to optimize exposure. Additionally, the inclusion of loading dose strategies supports a faster achievement of therapeutic levels, which may be particularly beneficial in the management of acute epileptic conditions. Overall, this model provides a robust framework to support evidence-based, patient-tailored dosing of PB, with the potential to enhance both treatment efficacy and safety in canine epilepsy management.

## Data Availability

The data used in this study are not publicly available due to confidentiality restrictions. However, they may be provided by the corresponding authors upon reasonable request and subject to confidentiality agreements.
